# User Evaluation of Passenger Assistance System Concepts on Public Highways

**DOI:** 10.3389/fpsyg.2021.725808

**Published:** 2021-12-09

**Authors:** Sandra Ittner, Dominik Mühlbacher, Alexandra Neukum, Thomas H. Weisswange

**Affiliations:** ^1^WIVW GmbH, Veitshöchheim, Germany; ^2^Honda Research Institute Europe GmbH, Offenbach, Germany

**Keywords:** discomfort, passenger, assistant system, human machine interface, real driving study, ADAS

## Abstract

There is ample research on assistance systems for drivers in conventional and automated vehicles. In the past, those systems were developed to increase safety but also to increase driver comfort. Since many common risks have by now been mitigated through such systems, the research and development focus expanded to also include comfort-related assistance. However, the passenger has rarely been taken into account explicitly, although it has been shown that passenger discomfort is a relevant problem. Therefore, this work investigated the potential of passenger assistance systems to reduce such discomfort. Three different passenger assistant system prototypes were tested in a driving study on public highway with *N* = 19 participants. The systems provided information about parameters related to the performance of the driver and one additionally provided a communicative means of influence. For two passenger assistant systems, it could be shown that they significantly reduced passenger discomfort in at least a subset of the evaluated situations. The majority of participants rated one or multiple of the assistant systems as more comfortable than a ride without assistance. The system providing information about the attentiveness of the driver was most effective in reducing discomfort and was rated as the most helpful system. The results show that explicitly considering the situation of passengers in the design of assistance systems can positively impact their comfort. This can be achieved using information from common systems targeting driver assistance available to the passenger.

## Introduction

The first driver assistance systems, such as the anti-lock braking system, appeared around 50 years ago (Gietelink et al., 2006; Galvani, 2019) with the main aim to increase safety by stabilizing vehicle dynamics. An early focus was put on safety problems related to extreme situations, but in the years that followed, improvements in regular driving situations became more and more important. Advanced driver assistance systems (ADAS) focus on increasing safety but also on increasing driver comfort by including often warnings or information toward the driver (e.g., Forward Collision Warning). Adaptive Cruise Control (ACC), for example, takes over continuous and attention-intensive driving tasks, such as keeping speed and the distance to a vehicle in front constant (Bengler et al., 2014). Means to increase vehicle safety and driver comfort through ADAS are already well researched; however, little focus was so far put on using related methods to also increase the comfort of passengers.

There has been little research focused on passenger discomfort in general or how to actively increase their comfort using assistant systems. One exception is research about driver comfort in the context of vehicle automation (Hartwich et al., 2018; Strauch et al., 2019; Hartwich et al., 2020). As the level of automation of a vehicle increases, the tasks and thus the control of the driver over the vehicle will decrease. As a result, the role of the driver will become increasingly similar to that of the passenger. Nevertheless, there are differences between a conventional passenger and the driver as a passenger of the automated vehicle. On lower SAE levels 3–4 (SAE International, 2014), the driver, unlike the passenger, still has the possibility to take over the control again in an emergency and also has information available, such as planned actions of the automated vehicle. On level 5 (SAE International, 2014) the positions are most similar. What does not matter in the context of automation, however, are social factors such as the relationship between driver and passenger as in a conventional vehicle. Another exception regarding research on passenger discomfort is research on the concept of motion sickness, in which symptoms are primarily triggered by physiological processes. The processes leading to motion sickness and its symptoms were described in the sensory conflict/rearrangement theories by Irwin (1881), Claremont (1931), or Reason (1978) and related theories (Bos and van der Bles, 1998; Bles et al., 1998). In the area of motion sickness, attempts are being made to prevent this with displays, among other things. Diels and Bos (2015) mention in their work design principles like display size, position, or information content that can be considered when designing user interfaces against motion sickness in automated vehicles. According to their work, displays with a small field of view, a display position along the line of sight, and using augmented reality should be selected to reduce motion sickness. In addition to physiological processes, there are also other processes that can cause passengers to feel uncomfortable. For example, social factors such as an unfriendly or unsympathetic driver. Cognitive and psychological mechanisms for discomfort that can arise when a passenger perceives a driving situation as unsafe e.g., during close following, are another field and will be addressed by the systems discussed in this paper.

Research about systems that take into account the role of the passenger often nevertheless evaluate those with a focus on the driver’s experience. In a study by Perterer et al. (2015), it was investigated whether the passenger can take over the navigation task for the driver to relieve him/her during the driving task. For this, the passenger had a detailed navigation system to provide relevant information to the driver. Maurer et al. (2014) proposed a prototype human-machine interface (HMI) which visualized the passenger’s gaze for the driver as a red point in a simulator scene. The visualized gaze of the passenger should make communication regarding directional information during navigation or upcoming hazards easier and more accurate which reduces misunderstandings. Trösterer et al. (2015) investigated the influence of this assistant system on the communication between driver and passenger in demanding situations in a simulator study. They also compared this visualization of the passenger’s gaze to a more reduced design, in which the gaze is visualized with LED lights. The LED lights were placed as a strip at the bottom of the windshield. In a user study with driver/passenger pairs, the results showed that the dot representation enabled the passenger to highlight things with higher accuracy in comparison to the LEDs. Passengers also indicated that they felt that the driver was less satisfied with the navigation information displayed with the LED system and found it less helpful. All presented studies have in common that, although, the passenger was taken into account in the design of the assistance systems and can also operate them, the information is still intended for the driver, while the passenger’s role is in supporting the driver. However, a survey has shown that passenger discomfort is a common problem (Ittner et al., 2020). Based on the results from this survey, a previous simulator study (Ittner et al., 2019, 2021) investigated different information concepts inspired by a cognitive model of passenger discomfort (Ittner et al., 2020) toward their potential benefit during driving. The results showed that it was possible to reduce passenger discomfort with certain concepts, while other concepts investigated in the study proved to be less suitable. Inspired by these concepts, three concrete co-driver assistance systems are developed in this paper and will be evaluated with users in a number of relevant realistic situations.

The evaluation will be performed using prototype systems in real vehicles in public traffic. Such a user study has a number of up- and downsides compared to a simulator study as can be seen from related ADAS work. Often concepts are tested in the simulator due to higher reproducibility caused by better controllability of environmental conditions. In simulator studies, however, there are also disadvantages such as a change in the assessment of hazardous situations due to a varying perceptibility of the situation (e.g., distance estimation) and a limited reproduction of the situation (e.g., acceleration, deceleration; Breuer, 2012). In field studies, environmental conditions or risk factors require more elaborate study designs, as these factors are more difficult to control. However, when any concrete ADAS functionality should be developed toward deployment or a high validity is important, real driving studies are often chosen (Breuer, 2012). Therefore, the concrete passenger assistance systems in this work will be evaluated in such a real-world user study.

The next section will shortly discuss the existing model on the processes leading to passenger discomfort. After that, three co-driver assistant systems are presented including their targets based on the cognitive discomfort model, related concepts from ADAS research, and the way they were implemented for the user study. In the second part of the work, the design of the public road user study is presented, in which the effect of the passenger assistance systems on their discomfort was examined. The paper will close with a discussion and an outlook on the future of “co-driver assistance systems.”

## Co-Driver Assistance Systems

In previous work (Ittner et al., 2020), a model was formulated which describes the possible mechanisms leading to passenger discomfort while driving. According to the model, there are two general mechanisms that can cause discomfort or prevent passengers from coping with it. Firstly, uncertainty about the driver’s cognitive state (for example, “How attentive is the driver?” or “How does the driver assess the situation?”) can lead to lowered confidence in the driver in certain situations and possibly lead to an assessment of a situation as more safety-critical, compared to the assessment of the driver. This assessment then causes discomfort. Secondly, passengers have limited or no possibilities to act, to prevent, or resolve potentially safety-critical situations. This can lead to the feeling of being exposed to the situation and cause high passenger discomfort. In a prior user study (Ittner et al., 2019, 2021), different information concepts, which were based on this model, were tested for their potential to reduce passenger discomfort in driving. The insights from this simulator study were used to design three different passenger assistant systems, which will be introduced in the following. Two of the assistant systems provide information about aspects of the driver’s cognitive state. The third assistant system additionally provides a limited means of control for the passenger.

### Shared Driver Attention System

The aim of this system was to provide both passenger and driver with feedback on the driver’s attentiveness or distraction from the driving situation. This feedback mainly aimed at reducing the passenger’s uncertainty about the driver’s assessment of the situation.

Driver states like drowsiness or attention are well researched in the field of driver assistance (Chacon-Murguia and Prieto-Resendiz, 2015) and already included in a number of commercial vehicles. As soon as fatigue or inattention is detected by these systems, the driver is warned visually and/or audibly (e.g., Shaout et al., 2011). Detection of inattention states can be done using different means, including driving behavior, physiological measures, and evaluation of driver features (Saini and Saini, 2014). One common approach is using the driver’s eye and head movements (Murphy-Chutorian and Trivedi, 2008). For example, Tawari and Trivedi (2014) evaluated the gaze of the driver in relation to the surrounding traffic. Toyota developed a Driver Monitoring System which also uses infrared cameras to investigate the driver’s head position (Toyota, 2005). This system was combined with their Advanced Pre-crash System which detected obstacles on the street. When the Pre-crash System detects an obstacle and at the same time the monitoring system detects that the driver has turned his head away from the road, a warning for the driver occurs. If he/she does not react, the brakes are activated.

Given the technical feasibility of driver attention estimation, it can be assumed that this information would also be available for passenger assistance systems. However, in contrast to the target of a driver assistance system, which should alert the driver only in case of inattentiveness, the proposed system will mainly aim to provide information about the continuous attentiveness of the driver. By providing this information *via* an HMI, a passenger might become more certain about the driver’s situation assessment, and additionally would be aware that the driver also would get feedback in case of distractions.

**Figure 1:**
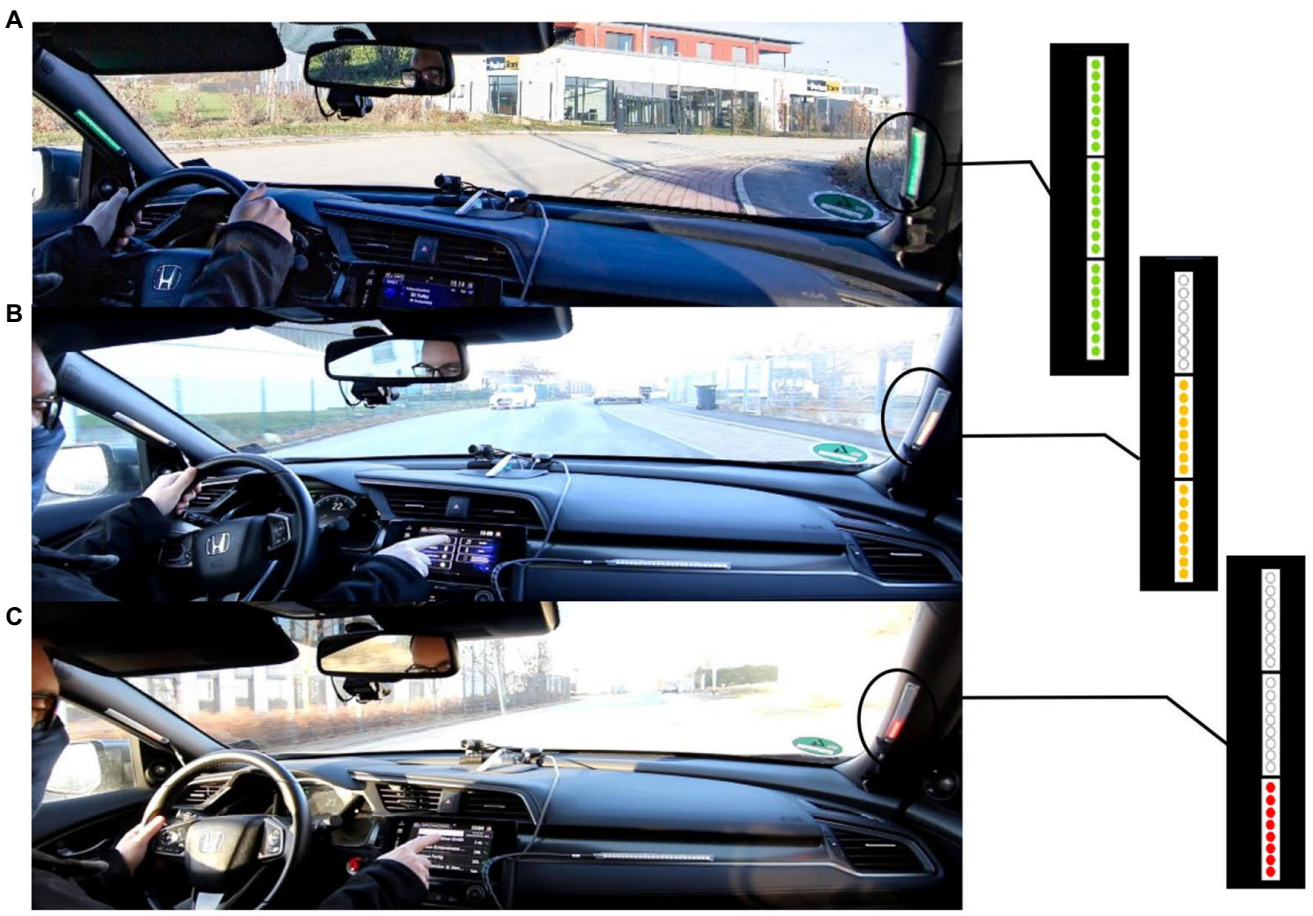
State transition of the LEDs strips communicating **(A)** high attentiveness, **(B)** medium, and **(C)** very low attentiveness as driver is performing a secondary task.

The first prototype for a passenger assistance system that is presented here displays the driver’s attention state to the passenger using a LED strip attached to both A-pillars (Figure 1). Each LED strip consists of three individual elements, each with eight LEDs on it. The state of the driver is communicated on one hand through colors and on the other hand through the number of lit LEDs. Three levels are distinguished for driver attention and matched to colors and a fixed number of lit LEDs. The number of lit LEDs mimics a percentage bar and the colors were selected from green to red as in a traffic light. When the driver is fully alert, all LEDs light up in green to signal to the passenger that the driver is concentrating on the driving situation with high attentiveness. When the driver is slightly distracted from the driving situation and is not permanently looking at the road (Figure 1B for example, while adjusting the radio), 2/3 of the LEDs are lit in orange. This condition shows the passenger that the attention is reduced. A state of increased distraction during prolonged gaze away from the road, for example, when the driver is looking at his cell phone, reading a message (Figure 1C), is visualized with red LED’s and only 1/3 of the LED strip is lit. This is supposed to signal that the driver’s attention is low.

### Shared Safety Distance System

The aim of this system was to show the passenger and driver the safety distance to the vehicle in front as an objective value. This could allow a passenger to ground his/her own assessment of the distance. At the same time, it might also have a positive effect that the driver has the same information and could be expected to react to small safety distances based on it. Even if the driver does not adapt to the distance in a certain situation, the passenger could interpret this as a confirmation that the driver considers the current distance as sufficient. In addition, the information could be used as an objective reference for communication to the driver that the safety distance might not be sufficient at a given time.

Adaptive Cruise Control is an ADAS available in many vehicles which already enables the driver to maintain a constant distance to a vehicle in front. Usually, it uses a sensor located around the front bumper to detect vehicles and then adjusts distance and speed automatically. The driver can usually select the desired distance out of a number of objectively safe options. However, the actual distance of a vehicle or the exact braking distance during approach are usually not displayed to the driver, and he/she therefore has to either trust the system to always be correct or learn over time at which distance the system starts reacting. This situation has some analogies to the situation of the passenger that does not know when a driver plans to brake and if he/she correctly perceived the actual distance. In addition, a passenger does not have a direct means to actually influence the braking distance. Israel et al. (2010) investigated an adaptation of the ACC concept. A contact analog display was used to present a bar visualizing the control criticality of the ACC. The bar had a red and green part and was displayed behind the target vehicle of the ACC. When the distance to the vehicle in front decreased and the own vehicle entered the red area of the bar, the ACC automatically started to brake and increase the distance again. In a field study, this ACC visualization was compared with a conventional visualization of an ACC in the instrument cluster. The participants showed no deterioration of their driving performance and an increased sense of security. The increased sense of security is explained by the authors as a result of additional information provided by the display.

**Figure 2:**
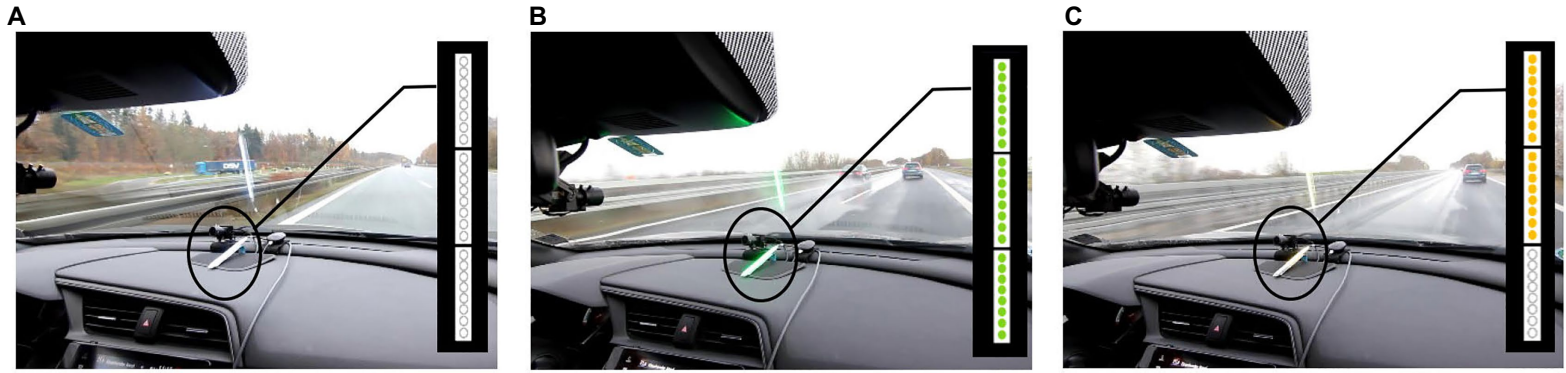
Transitions of the Shared Safety Distance (SSD) system from **(A)** no car is detected over **(B)** green when a car in front is detected with sufficient safety distance to **(C)** orange when distance is below the safety threshold.

Information from existing systems such as the ACC could also be used to provide help against distance-related discomfort for the front passenger. The second proposed passenger assistance system presents both driver and passenger with information about the safety distance to the vehicle in front. To be more precise, it provides information about the time headway (THW) to the car in front, which is taking into account speed differences. As with the previous system, an LED strip consisting of 24 individual LEDs is used. The strip is mounted at the center of the dashboard parallel to the direction of travel (Figure 2). The distance to the front car is represented by the number of LEDs that are lit (for closer cars the LEDs at the farthest distance to the driver start to turn off). Additionally, a color-coding emphasizes the “safety” of a given headway. White illumination indicates that the system is active, but no preceding vehicle is detected (Figure 2A). If a vehicle in front is detected by the system and the distance is sufficient, taking into account the current speed difference (THW > 1.2 s), all LEDs light up and are green. This signals to the driver and passenger that at a headway of 1.2 s or more the driver could still react and prevent an accident in case of sudden strong braking of the front vehicle. If the THW to the front vehicle falls below 1.2 s, the LEDs change their color to orange and only 2/3 of the LEDs light up, showing the reduced THW (Figure 2C). This signals to the driver and passenger that the current headway should be increased to guarantee safety. As soon as the headway is reduced even further (THW < 0.8 s), the LEDs light up red and only 1/3 of the LED stripe remains lit. This state of the system shows the driver and passenger that it would no longer be possible for the driver to react in time.

### Adaptable Shared Safety Distance System

This system should offer the passenger the possibility to provide input when the perceived distance is starting to feel uncomfortable. Usually, a passenger can cope with perceived safety-critical situations only in an emotion-focused way, for example, by distracting him/herself. Problem-focused handling is often not possible since the passenger has no control over the vehicle. There is only the indirect possibility to ask the driver for a change of behavior, for example, to increase the distance to a front vehicle. However, the effect of such coping depends on the driver’s compliance with this request and his/her character. A possibility to intervene using a less subjective means could reduce the passenger’s feeling of being exposed to safety-critical situations.

**Figure 3:**
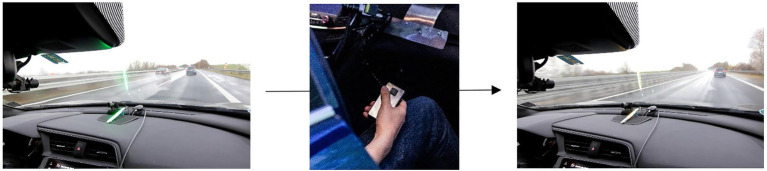
Color transition from green to orange caused by a button press of the passenger.

However, as providing a passenger means of influencing the actual driving behavior might be dangerous, the third proposed passenger assistance system acts through an objective reference shared between driver and passenger. This information is represented by the THW as in the Shared Safety Distance (SSD) system, also using the same basic HMI setup. However, the passenger can influence the color of the LEDs him/herself by pressing a button to signal that the current distance does not feel comfortable. A button-press switches green LEDs to orange (Figure 3). With a push of the button, the passenger can only set the displays of the SSD system to a more safety-critical level, but not the other way around. This means also that the passengers are not forced to press the button or to pay attention all the time.

The three presented assistance systems [Shared Driver Attention System (SDAT), SSD, and Adaptable Shared Safety Distance System (ASSD)] will be evaluated in a real-world context with respect to their general potential to reduce discomfort in passengers and to some effects specific to the underlying cognitive concepts addressed.

The following hypotheses will be tested:

*H1a*: With assistance systems, situations that cause discomfort are assessed as less uncomfortable than without assistance.

*H1b*: With assistance systems, an entire ride is subsequently assessed as less uncomfortable than without assistance.

*H2*: With assistance systems, situations that cause discomfort are assessed as less critical than without assistance.

*H3*: In situations that cause discomfort, the SDAT assistance reduces uncertainty on whether the driver is attentive compared to the unassisted case.

*H4*: In situations that cause discomfort, the SSD assistance reduces uncertainty on whether the driver correctly assessed the situation compared to the unassisted case.

*H5*: In situations that cause discomfort in the baseline condition, the ASSD assistance reduces the feeling of being exposed compared to the unassisted case.

The aim of this work was to investigate if the passenger assistance systems could improve passenger experience in uncomfortable situations. The experiments are performed under the assumption that only if a person experiences at least slight discomfort in a given situation, it would be expected that such an effect would be visible. They were therefore set up in a way to focus on participants and situations for which discomfort is a problem. We also assume that it is possible to create repeatable situations under real traffic conditions in which passengers feel uncomfortable although the situations are objectively safe.

## User Study

### Methods

#### Sample

In the study, *N* = 19 participants (*n* = 6 male; *n* = 13 female) took part with an age between 20 and 80 years (*m* = 41.3 years; *SD* = 19.7 years). There were no special exclusion criteria except for passengers who generally reported to not get nervous very easily. This information was gathered by means of a preliminary survey. In this survey, potential participants answered the question “I feel uncomfortable as a passenger faster or more often than most other passengers” and “How nervous do you consider yourself to be as a passenger?” on a 16-point category subdivision scale. Every participant who answered one of the two questions with a rating of 7 or above was invited to the study.

#### Procedure

The study was approved by the institutional ethics committee at the WIVW GmbH. This ethics committee follows recommendations of the German Research Association (Deutsche Forschungsgemeinschaft, 2019). Participants experienced a test drive, consisting of four rides on a German autobahn, in a commercial vehicle as a front-seat passenger. The starting point of the first ride was at a commuter parking lot near Würzburg Kist, Germany. The route followed the A 81 highway in the direction of Stuttgart up to a second commuter parking lot (Ravenstein) 50 km away. The autobahn has mostly two lanes and no speed limits. During the short parts, where the highway had more than two lanes or a speed limit was present no experiment situations were set up. The second ride followed the inverse route back to Würzburg Kist. These two routes were repeated for a third and fourth experiment session. In three of the four rides, one of the passenger assistant system variations was activated and the remaining ride without a passenger assistant system was used as a baseline. Before each ride with an assistance system, the subjects received written instructions explaining the function of the respective system. In addition, they were shown a demonstration video in which all system states were presented. During a ride, participants experienced a number of different situations which used a specific setup (see “Situations”) to allow for reproducible situation type and order. The individual rides took approximately 30 min. Between each ride, there were short breaks with a post inquiry of approximately 10 min. The overall study duration was around 3 h. The driver was an employee of the WIVW with a test driver license and was previously familiarized with the situations (including when to interrupt the testing, compliance with road traffic regulations, etc.). The driver of the test vehicle was introduced to the participants as also being a study participant to prevent biased expectations. To create more realistic experimental conditions, the driver also pretended to fill in the questionnaires after each ride. After finishing the study, participants were informed about the cover story.

During most of the experiment, the driver was instructed to use the ACC functionality and the lowest setting of 1.2 s (according to the car’s manual) to any front vehicle to guarantee a stable, safe, and reproducible headway. The ACC was only deactivated during overtaking maneuvers and when exiting the highway. The driver was also instructed to keep his eyes on the road. Both instructions aimed at creating situations without an objective/safety reason to feel discomfort. The passenger was not informed that the driver had received these instructions. As the main interest was to evaluate the systems’ potential to reduce discomfort caused by the core mechanisms explained in the previous section, the participants always received positive feedback during all rides with a system. During the test scenarios with the SDAT system, for example, participants exclusively saw the green LED’s. This was also the case for the other two systems. Only during overtaking maneuvers, the color of the LEDs could briefly change to orange as the safety distance was often undercut.

On the rear seats, an experimenter was placed who controlled the assistance systems (wizard-of-oz method) and gave instructions to the participant when he/she had to evaluate situations. The position of the experimenter in the back seat allowed him/her to see the ACC display and use it to adjust the settings of the SSD and ASSD system according to it. If no vehicle was detected by the ACC, the experimenter set the system to white to indicate that the SSD/ASSD system did not detect one either. As soon as a front vehicle was detected by the ACC, the experimenter also set the white LEDs to green to signal to the participant that the safety distance was sufficient. The wizard-of-oz setting was chosen to ensure reproducibility during all rides and allowed the use of a standard production vehicle.

#### Situations

During each ride, participants experienced seven different situations which are described in detail in Table 1. In a previous survey (Ittner et al., 2020), close following was most frequently mentioned as a situation in which passengers feel uncomfortable, therefore a majority of selected situations relate to this. The situations were also selected to be frequently occurring when driving on the autobahn. Multiple different variations were selected to increase the probability of finding situations that cause discomfort for at least some of the participants.

**Table 1:** Description of situations performed during each ride.

Approaching	Lane change confident	Indicated lane change confident	Lane change ego	Overtaking	Exiting	Entering
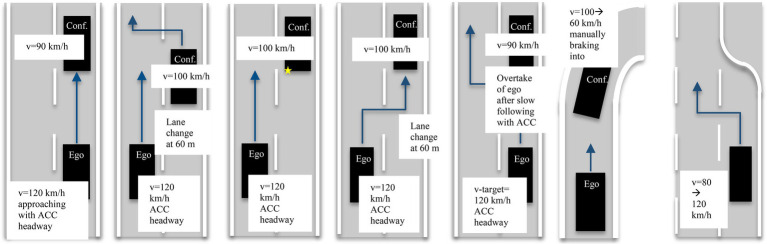						

The SDAT system information can be relevant in all situations shown. The safety distance information is not relevant in situations of type “LC Indicated” or less relevant when “Entering” the autobahn if there is no vehicle in front or on the target lane. The order of the situations was randomized for each ride to avoid learning effects. To keep risks for the participants and other road users low, there were different safety and control measures for the execution of the situation defined. Driving speed on the Autobahn was set to a maximum of 120 km/h. Additionally, test drives were postponed or interrupted at very high traffic density, in case of poor visibility (e.g., fog), or slippery road conditions caused by rain. A situation was aborted if there was insufficient distance to other vehicles, for example, after a cut in. To guarantee a number of relevant situations and to allow situations from multiple runs to be as similar as possible, a second vehicle (“confident” vehicle), operated by another trained experimenter was participating in the test drives. Before the start of the procedure, participants received a written and oral explanation of the general procedure, including that they could stop the experiment at any time without explanation if the situations are perceived as too stressful and gave their informed consent. In all of the situations, the driver reported no feeling of discomfort.

#### Dependent Variables

**Figure 4:**
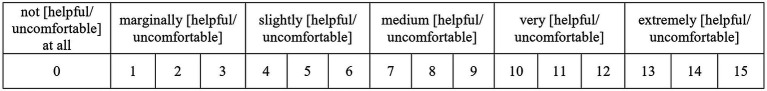
Sixteen-point category subdivision scale used for the rating of system or situation after each situation and in the post inquiry for the example of “helpfulness” or “uncomfortable.”

After each situation, participants used a tablet to rate on a 16-point category subdivision scale (Heller, 1985; Figure 4; “online ratings”):

- The intensity of their experienced discomfort.
- The criticality of the situation.
- How uncertain they were about where the driver’s attention was.
- How uncertain they were as to whether the driver had correctly assessed the situation.

After four trips, there was a post inquiry in which the participants rated how helpful each assistant system was in reducing their discomfort (16-point category subdivision scale). They also reported a ranking of the four rides from most comfortable to least comfortable.

#### Situation Discomfort Rating

**Figure 5:**
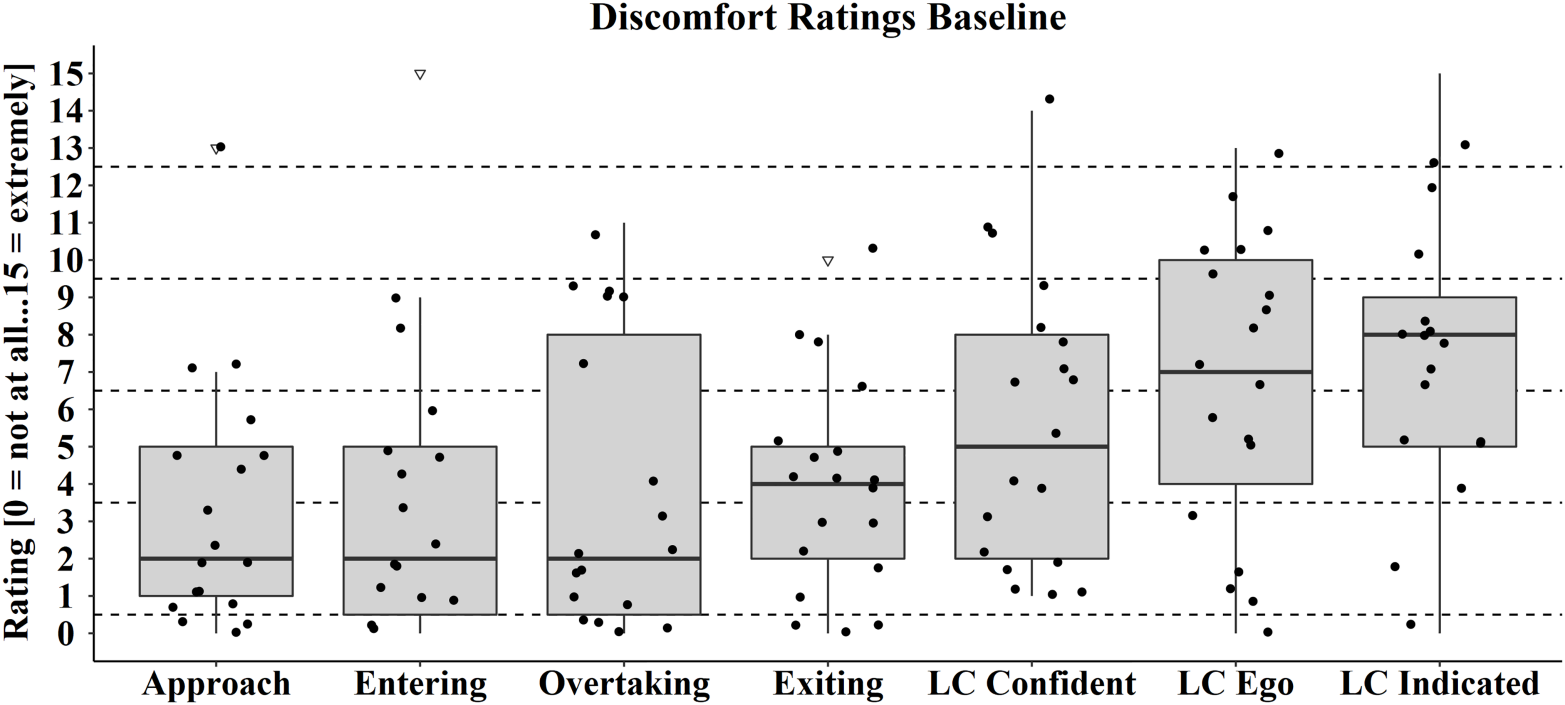
Discomfort ratings for each situation in the baseline ride. The dots represent the individual discomfort ratings of each participant in the situations. Box range = Q1 to Q3. Whiskers = 1.5 ^*^IQR.

Even without any assistance systems, the selected situations were evaluated differently by the participants with respect to discomfort induction (Figure 5). “Approach” and “Entering” situations showed the lowest discomfort ratings, followed by “Overtaking” and “Exiting.” Participants felt most uncomfortable during the situations involving lane changes (LC).

Despite the pre-selection of participants based on the two questions in the pre-survey, there were two participants [test subject (TS) 1 and 2] who experienced no or marginal discomfort in all baseline situations (Table 2). TSs 3–9 experienced between 2 and 4 situations in which they felt at least slight discomfort. Two situations of TS 2 (one exiting in the ASSD ride and one LC indicated in the SSD ride) could not be used for evaluation because they could not be successfully performed.

**Table 2:** Descriptive statistics for the baseline discomfort ratings (arranged by median value) and total number of tested situations per participant.

TS	Mean baseline discomfort rating	SD baseline discomfort rating	Median baseline discomfort rating	Total number of situations tested in the study per participant
1	0.29	0.49	0	28
2	0.57	1.13	0	26
3	5.29	5.56	1	28
4	3.86	5.05	2	28
5	3.00	1.77	2	28
6	2.00	2.34	2	28
7	3.00	1.92	2	28
8	2.86	1.95	3	28
9	3.71	2.14	4	28
10	5.00	4.24	5	28
11	5.14	2.04	5	28
12	5.86	2.91	7	28
13	5.00	3.42	7	28
14	7.57	3.87	8	28
15	6.86	2.12	8	28
16	7.71	4.23	8	28
17	7.29	4.92	9	28
18	8.57	4.69	9	28
19	8.43	2.57	9	28

### Results

#### Online Ratings

For the analysis of the hypotheses non-parametric dependent Wilcoxon tests (one-sided) were used to address the relatively low individual sample sizes.

**Figure 6:**
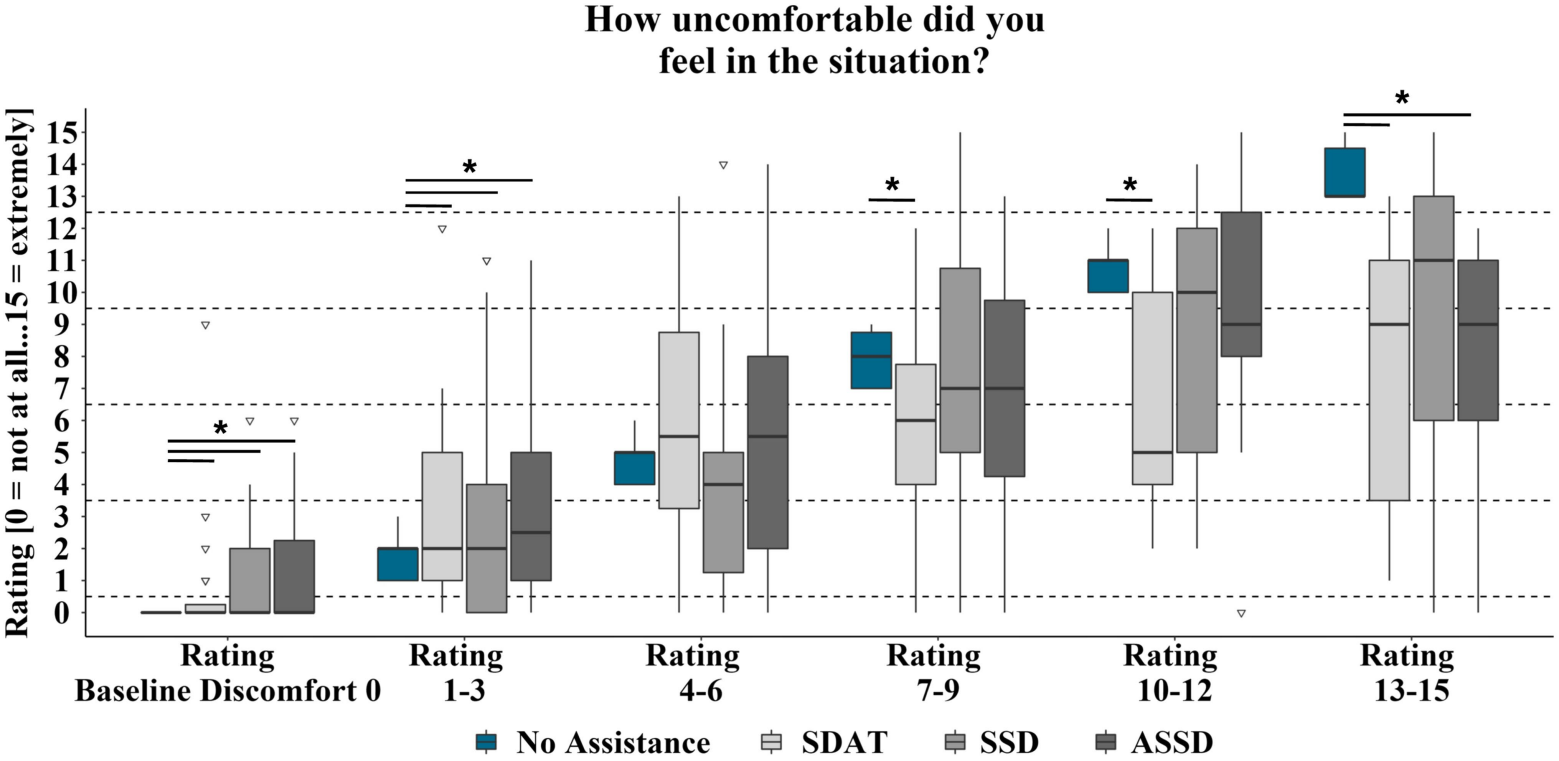
Discomfort ratings for rides with and without assistance by discomfort ratings in the baseline ride. Significant (*p* < 0.05) differences between ratings with and without assistance system are marked with *. Box range = Q1 to Q3. Whiskers = 1.5 ^*^IQR.

The first hypothesis (*H1a*) was that the assistant systems would significantly reduce or prevent discomfort that was experienced in the respective baseline situation. If all uncomfortable situations were considered (all baseline discomfort ratings > 0), there was no assistant system which significantly reduced passenger discomfort (SDAT: *N* = 113, z = −1.58, n.s.; SSD: *N* = 113, z = −0.85, n.s.; ASSD: *N* = 112, z = −0.27, n.s.). As has been stated before, it was assumed, however, that only above a certain level of discomfort the assistance systems would have a reducing effect. Therefore, in the following evaluations the discomfort reduction of the individual assistance systems is investigated separately for all baseline discomfort categories on the scale. Table 3 and Figure 6 show that for all three assistance systems there was even an increase of passenger discomfort for the baseline discomfort categories in which the passengers experienced no or marginal (1–3) discomfort. The assistant systems showed no effect in the slightly uncomfortable (4–6) baseline situations category. When the discomfort level in the baseline situations reached medium (7–9) discomfort there was a significant reduction of the passenger’s discomfort by the assistant systems SDAT. This system also generally showed the strongest reduction of discomfort across the higher categories. The ASSD system only reduced discomfort in situations experienced in the ride without assistance as extremely uncomfortable (13–15). The SSD system showed no discomfort reducing effect. An evaluation of the individual assistance systems’ effect in the respective situations is not possible with this approach, as otherwise the group sizes would become too small or, in some cases, no more data points would be available for each situation. Spearman correlations showed significant relations between the participants’ discomfort reductions by each assistant system and their discomfort ratings in the ride without assistant system (SDAT: *N* = 133; *r* = 0.45; *p* < 0.001; SSD: *N* = 132; *r* = 0.31; *p* < 0.001; ASSD: *N* = 132; *r* = 0.35; *p* < 0.001). This means that with higher discomfort ratings the assistant systems showed a stronger reduction.

**Table 3:** Exact Wilcoxon-Tests (one-sided) for discomfort rating differences between assisted and non-assisted rides by assistant system and by scale category of the baseline discomfort ratings.

Scale category		Baseline discomfort	SDAT discomfort	SSD discomfort	ASSD discomfort
Baseline discomfort ratings 0	mean	0	0.80	1.21	1.30
	N	20		19	20
	z	−2.03		−2.68	−2.53
	*p*	0.031		0.002	0.004
	*η^2^*	0.206		0.378	0.32
Baseline discomfort ratings 1–3	mean	1.79	2.77	3.29	3.08
	N	39		39	38
	z	−2.14		−1.79	−2.61
	*p*	0.016		0.037	0.004
	*η^2^*	0.117		0.082	0.179
Baseline discomfort ratings 4–6	mean	4.73	5.85	4.04	5.54
	N	26		26	26
	z	−1.41		−1.24	−0.80
	*p*	n.s.		n.s.	n.s.
	*η^2^*	–		–	–
Baseline discomfort ratings 7–9	mean	7.90	5.77	7.30	7.20
	N	30		30	30
	z	−2.83		−0.62	−0.81
	*p*	0.002		n.s.	n.s.
	*η^2^*	0.267		–	–
Baseline discomfort ratings 10–12	mean	10.73	6.91	8.91	9.27
	N	11		11	11
	z	−2.45		−1.47	−0.98
	*p*	0.008		n.s.	n.s.
	*η^2^*	0.546		–	–
Baseline discomfort ratings 13–15	mean	13.71	7.43	9.14	7.86
	N	7		7	7
	z	−2.20		−1.53	−2.37
	*p*	0.016		n.s.	0.008
	*η^2^*	0.691		–	0.802

n.s., not significant *p* ≥ 0.05.

**Figure 7:**
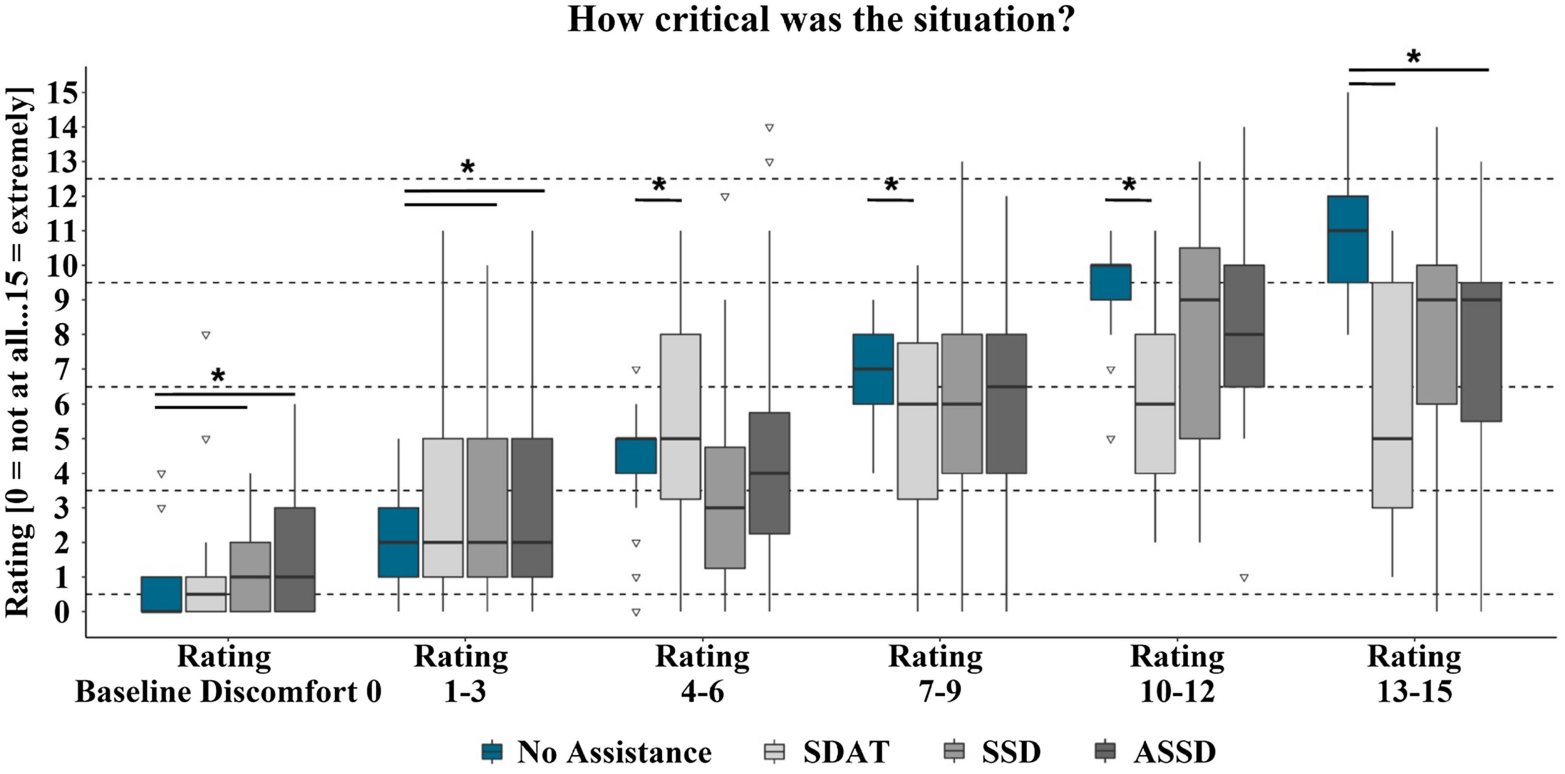
Criticality ratings for rides with and without assistance by discomfort ratings in the baseline ride. Significant (*p* < 0.05) differences between ratings with and without assistance system are marked with *. Box range = Q1 to Q3. Whiskers = 1.5 ^*^IQR.

The second hypothesis (*H2*) was that situations would be assessed as less critical when the information provided by one of the assistance systems is available. Again, situations in which the participants experienced no or marginal discomfort in the baseline condition were rated as more safety critical when experienced with the SSD and ASSD system (Table 4 and Figure 7). For the SDAT system, this was also the case for situations in which the passengers felt slightly uncomfortable during baseline drives. For other uncomfortable situations, the SSD system showed no influence on the safety critical ratings. Situations that were rated by the passengers as at least medium uncomfortable without assistant system were rated less safety critical with the available information of the SDAT system. For the ASSD system, this was only true for situations that were experienced as extremely uncomfortable without assistant system. Spearman correlations showed significant relations between the participants’ discomfort and criticality ratings in the ride without assistance (*N* = 133; *r* = 0.92; *p* < 0.001).

**Table 4:** Exact Wilcoxon-Tests (one-sided) for criticality rating differences between assisted and non-assisted rides by assistant system and by scale category of the baseline discomfort ratings.

Scale category		Baseline criticality	SDAT criticality	SSD criticality	ASSD criticality
Baseline discomfort ratings 0	mean	0.60	0.80	1.05	1.65
	N	20		19	20
	z	−1.04		−1.99	−2.05
	*p*	n.s.		0.031	0.021
	*η^2^*	–		0.208	0.210
Baseline discomfort ratings 1–3	mean	2.15	2.74	3.21	3.26
	N	39		39	38
	z	−1.28		−1.98	−2.20
	*p*	n.s.		0.023	0.014
	*η^2^*	–		0.101	0.127
Baseline discomfort ratings 4–6	mean	4.27	5.54	3.73	4.62
	N	26		26	26
	z	−1.88		−1.26	−0.14
	*p*	0.030		n.s.	n.s.
	*η^2^*	0.136		–	–
Baseline discomfort ratings 7–9	mean	6.90	5.40	5.87	5.93
	N	30		30	30
	z	−1.98		−1.26	−1.54
	*p*	0.024		n.s.	n.s.
	*η^2^*	0.131		–	–
Baseline discomfort ratings 10–12	mean	9.27	6.18	8.00	8.27
	N	11		11	11
	z	−2.25		−0.87	−1.07
	*p*	0.012		n.s.	n.s.
	*η^2^*	0.460		–	–
Baseline discomfort ratings 13–15	mean	11.00	6.00	7.86	7.43
	N	7		7	7
	z	−2.37		−1.36	−1.95
	*p*	0.008		n.s.	0.031
	*η^2^*	0.802		–	0.543

n.s., not significant *p* ≥ 0.05.

Hypotheses 3–5 addressed the specific effects of each assistance system in situations causing discomfort during baseline driving. The SDAT assistant system significantly reduced the uncertainty of the participants whether the driver’s attention was on the situation in situations which were rated as at least medium uncomfortable in the ride without assistant system (*H3*) (Table 5). The SSD system did not reduce the passengers’ uncertainty whether the driver had correctly assessed the situation (*H4*). Similar to the discomfort and criticality ratings it even increased this uncertainty in situations rated as marginally uncomfortable in the baseline ride. The ASSD system reduced the passenger’s feeling of being exposed to a situation, in situations rated as at least medium uncomfortable in the baseline ride (*H5*).

**Table 5:** Exact Wilcoxon-Tests (one-sided) for differences in the specific effects of the assistances systems between assisted and non-assisted rides by scale category of the baseline discomfort ratings.

Scale category		Baseline – uncertainty attention	SDAT – uncertainty attention	Baseline – uncertainty correct assessment	SSD – uncertainty correct assessment	Baseline – feeling of being exposed	ASSD – feeling of being exposed
Baseline discomfort ratings 0	mean	0.05	0.20	0.11	0.32	1.45	2.15
	N	20		19		20	
	z	−1.13		−0.71		−1.46	
	*p*	n.s.		n.s.		n.s.	
	*η^2^*	–		–		–	
Baseline discomfort ratings 1–3	mean	0.95	1.00	1.33	2.59	4.39	4.29
	N	39		39		38	
	z	−0.22		−1.88		−0.07	
	*p*	n.s.		0.030		n.s.	
	*η^2^*	–		0.091		–	
Baseline discomfort ratings 4–6	mean	2.50	1.77	3.50	3.38	6.73	6.92
	N	26		26		26	
	z	−1.46		−0.44		−0.52	
	*p*	n.s.		n.s.		n.s.	
	*η^2^*	–		–		–	
Baseline discomfort ratings 7–9	mean	3.50	1.47	5.47	5.40	9.13	7.27
	N	30		30		30	
	z	−3.15		−0.46		−2.23	
	*p*	0.000		n.s.		0.012	
	*η^2^*	0.331		–		0.166	
Baseline discomfort ratings 10–12	mean	7.36	1.91	9.91	7.27	12.55	9.45
	N	11		11		11	
	z	−2.71		−1.37		−2.40	
	*p*	0.002		n.s.		0.008	
	*η^2^*	0.668		–		0.524	
Baseline discomfort ratings 13–15	mean	8.71	3.29	10.71	8.43	14.14	9.14
	N	7		7		7	
	z	−1.99		−1.11		−2.21	
	*p*	0.031		n.s.		0.016	
	*η^2^*	0.566		–		0.698	

n.s., not significant*p* ≥ 0.05.

#### Post Inquiry

**Figure 8:**
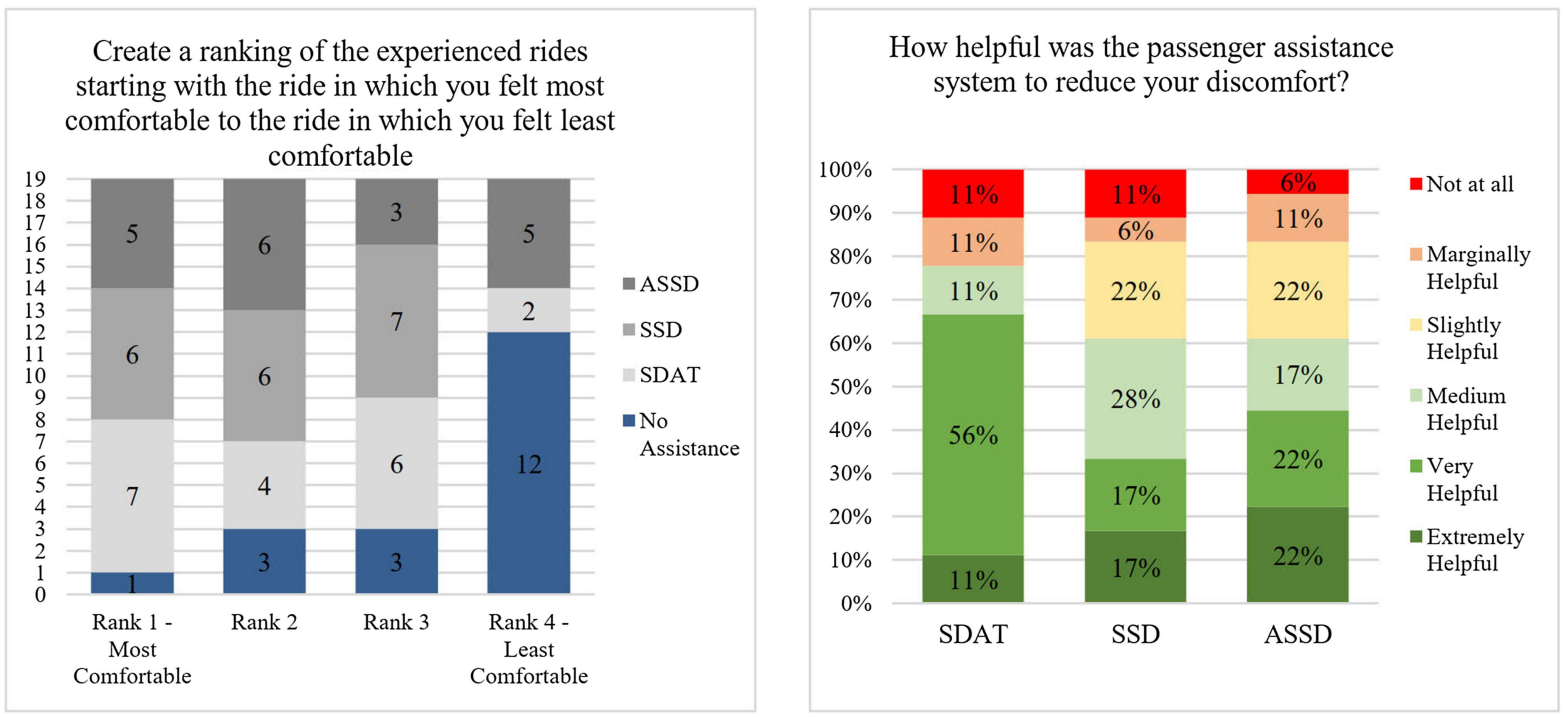
**Left:** Ranking of the assistance system rides from most to least comfortable. **Right:** Helpfulness ratings for the different assistance systems made in the post inquiry.

Figure 8 left shows the ranking of the rides from most to least comfortable (*H1b*). Only one participant felt more comfortable in the ride without any assistant system, while the other *N* = 18 participants felt most comfortable with one of the three assistant systems. Most of them preferred the SDAT system followed by the SSD and ASSD system. *N* = 12 participants felt least comfortable without any assistant system. With *N* = 5 participants feeling least comfortable with the ASSD system, followed by the *N* = 2 participants with the SDAT system.

The participants also rated how helpful the systems were to reduce their discomfort (Figure 8 right). The attention system was the best rated system. Around 67% of the participants rated the system as very or extremely helpful in reducing their discomfort. Additionally, 11% rated the system as medium helpful. Participants often mentioned during the post inquiry the positive effect that the attentiveness of the driver is visible, and that it is reassuring to be able to see this (Supplementary Table A. 1). However, there were also participants who mentioned that they do not need such a system for everyday use. The other two systems were rated slightly less helpful than the SDAT system. The distribution of the ratings shows that approx. about 34% of the participants rated the SSD system as very or extremely helpful to reduce their discomfort as did 44% with the ASSD system. The 28% of participants who rated the system as medium helpful for the SSD system were better than the 17% for the ASSD system. With the SSD system, the participants most often found it positive that one feels more secure with the system and is also more attentive in the situation (Supplementary Table A. 2). In addition, it was mentioned that an objective measurement is positive, and that one can compare one’s own subjective estimation with it. However, it was also negatively noted that the system is too insensitive and the distance to the vehicle in front is too small before the system changes to the orange state. For the ASSD system, it was also positively highlighted that by displaying the distance, it can be better estimated, the system makes you feel more comfortable, and that the button provides even more control (Supplementary Table A. 3). However, participants also mentioned that it takes some effort to press the button and that it provides a potential for conflict. About 11%, found the SDAT and SSD systems not helpful at all. This rate was lower for the ASSD system (6%).

### Discussion

Analysis of discomfort ratings at baseline showed that it was possible to create repeatable situations in real traffic, in which passengers feel uncomfortable in order to investigate the effect of the assistance systems. However, not all tested situations were equally likely to produce discomfort in participants. The participants’ mean discomfort ratings across all baseline situations showed that despite the pre-selection of nervous passengers, there were some who experienced only marginal to no discomfort in any situation. The lane change situations were the most likely to make participants feel uncomfortable. Other situations were less effective in producing stronger discomfort. This could be because lane change situations are much more dynamic and riskier compared to the other situations. Despite this fact, there were enough participants for these less effective situations who experienced at least some discomfort in these situations to investigate the effect of the assistance systems in these situations as well.

According to the co-driver discomfort model (Ittner et al., 2020), a change in the estimation of the situation criticality should reduce the passenger’s discomfort. With the information or control provided by the systems SDAT and ASSD, the passengers did indeed experience less discomfort with the largest effect for the SDAT system. The information of the SSD system alone showed no discomfort reduction. Only the combination of the SSD information and the possibility to signal the driver that the distance is too small for the passenger in the ASSD system showed a reduction of discomfort in situations rated as extremely uncomfortable in the baseline. One explanation could be that for some situations the THW presented as safe by the SSD system was too small for some participants to trust the information in these cases alone. This was also mentioned by some participants as a negative aspect of the SSD system in the post inquiry. The additional possibility of the ASSD system to signal a too small distance to the driver could have made a difference. The further evaluation showed that participants, who generally rated more situations in the baseline as uncomfortable, also experienced stronger discomfort than the other participants. It is possible that these more sensitive participants require a larger safety distance to feel comfortable and relaxed. As it was suspected in the introduction, the results also indicate, that the systems are more helpful and support front-seat passengers when situations are at least medium uncomfortable. In situations with less discomfort the systems showed no influence which could be caused by the fact, that the passengers did not need the information or control by the systems. In the marginal discomfort category, the assistant systems seemed to even slightly increase discomfort, although it always stayed within the marginal category. Since the experiment was not designed to investigate an increase in discomfort, it is unclear whether the effect was really caused by the system. The subjective participant ratings in the post inquiry showed that the systems were considered helpful in reducing discomfort (*H1b*). Apart from one participant, everyone felt more comfortable with one of the assistance systems than without. Most of them preferred the SDAT system closely followed by the SSD and the ASSD system. The participants also rated the SDAT system as most helpful in reducing their discomfort. These results confirm hypotheses 1*a* for the SDAT and ASSD system and *1b* for all systems.

According to the model, uncertainty about the driver’s cognitive state can lead to lowered confidence in the driver in certain situations and possibly lead to an assessment of a situation as more safety-critical which causes this discomfort. In the second hypothesis, it was therefore investigated if information about the cognitive state of the driver can reduce a safety critical assessment by passengers. In accordance with the discomfort model, the pattern in which situations the passengers felt less uncomfortable was similar to the situations that were assessed as less critical. This connection between the assessment of a situation’s criticality and experienced discomfort is also supported by the found relations. With the assistant systems SDAT and ASSD, participants rated situations as less safety critical than without the information displayed by the assistant systems. Again, this influence was found for situations that were rated as at least medium uncomfortable in the ride without assistance. For slight discomfort, SSD and ASSD increased the criticality, which is also similar to the increase in the discomfort ratings. All in all, the results confirm hypothesis 2 for the assistant systems SDAT and ASSD.

Hypotheses 3–5 investigated assistant system-specific effects postulated by the co-driver discomfort model. The SDAT and the SSD system should reduce uncertainties regarding the cognitive state of the driver. The ASSD system should additionally reduce their feeling of being exposed to a situation or, respectively, should reduce their feeling of not being able to intervene in such situations. With the SDAT system, the participants were less uncertain whether the driver was attentive during the tested situation if it was a situation, in which passengers experienced at least medium discomfort in the baseline ride. At the lower discomfort ratings, the system showed no reduction of uncertainty. Considering the average level of uncertainty in the baseline ride, this could be explained by the fact that the information is not needed, as the discomfort was also low. Hypothesis (*H3*) could therefore be confirmed.

Hypothesis (*H4*) cannot be confirmed. The SSD system could not reduce the uncertainty of whether the driver had assessed the situation correctly. For situations in which participants experienced marginal discomfort in the baseline uncertainty even increased, although this uncertainty was still marginal. Since the overtaking maneuver was the only situation, in which the system indicated that the safety distance was not respected, it is possible that the participants were more uncertain about this information causing an increase in the criticality assessment and discomfort. This result thus also indicates that making a safety-critical situation explicit through the system may even increase the passenger’s discomfort compared to driving without this information. It should be further investigated if this has a negative influence on the acceptance of such passenger assistance systems or if it even increases safety. As drivers may then be more considerate to avoid such objectively safety-critical situations in order not to maintain the passenger’s well-being.

The button feature of the ASSD system could make the passengers feel less exposed during at least medium uncomfortable baseline situations (*H5*). However, this only reduced their discomfort and the criticality assessment in the extremely uncomfortable baseline situations. Even with the system, subjects still felt at least moderately exposed to the situations. One explanation arises from the post inquiry, where some participants mentioned that they hesitated to use the button. The system might not have provided an additional means compared to talking to the driver. Thus, the results partly confirm hypothesis 5.

The results of hypotheses 3–5 also explain the systems’ different influences on the criticality estimation and experienced discomfort found in the tests for the first two hypotheses. According to the model, it is assumed that, due to the missing information about the cognitive state of the driver, the criticality of the situations is estimated higher by the passenger than by the driver. In addition, the lack of possibility to intervene leads to the fact that the discomfort remains stable or increases (Ittner et al., 2020). This effect was most clearly visible with the SDAT system. With this system, clarity regarding the driver’s cognitive state was highest. Due to this, situations were assessed as less critical by the passengers and less discomfort was felt for situations that were rated as at least medium uncomfortable in the baseline ride. The information provided by the SSD system did not contribute to a lower criticality estimation of the situations and thus also did not lower discomfort for those. As the participants mentioned in the post inquiry it is possible that the presented THW was too small for the participants. As a result, the influence on the assessment of the situation’s criticality was also smaller and discomfort was reduced to a lower extent than in the SDAT system. The ASSD system also reduced the passengers’ feeling of being exposed but this only reduced the criticality assessment and discomfort in extremely uncomfortable situations. The medium exposed ratings in these categories indicate that the system can reduce the exposed feeling but only to a certain extent. Even with this system, the passenger does not have the full control that the driver does. These processes are also reflected in the subjective evaluations of the systems. The SDAT system was rated as the most helpful system to reduce discomfort and most of the participants experienced their most comfortable ride with it. The proposed systems usually showed a reducing effect on the higher base discomfort. This is also supported by the relationships between the strength of the reductions and the discomfort ratings in the baseline rides. However, this also indicates that there are probably more subtle or diverse factors than those addressed by our systems responsible for the lower discomfort feelings.

The previous simulator study (Ittner et al., 2019, 2021) investigated basic concepts that are reflected in the systems proposed in this work. The aim of the simulator study was to test first concepts of passenger assistance systems to see if it is possible to reduce discomfort with them and if they are suitable as assistance systems. In the simulator study, the concept that visualized driver attention was rated best followed by the concept that visualized the objective distance and the influence on the distance concept. However, the concept that visualized the objective distance was rated as more helpful than the influence on distance concept. The simulator study concepts that were most suitable were investigated in this work in a real driving context. Thus, it was possible to investigate their influence on discomfort also under real-world conditions. In absolute terms, all systems were rated slightly less helpful in the post inquiry of the real driving study. This may possibly be due to a change in technical implementation. In the real driving study, the ASSD system combined the functions of the concept that visualized the objective distance and the influence on distance one from the simulator study. The ratings found for real traffic were between those two concepts. In the simulator study, the distance information, which was here used for SSD and ASSD, was projected onto the road in front of the ego vehicle by means of a head-up display, which might have made the information more intuitively understandable and easier to use and could explain the slightly higher results. Similar to the simulator study, there was a discrepancy between the discomfort ratings in the situations and how helpful the systems were rated in the post inquiry. In the inquiry, most subjects rated the systems as helpful and preferred them compared to driving without an assistance system. However, a discomfort reduction could not be found with every system and in all situations. These results indicate that the systems are more likely to have a comprehensive effect on discomfort than a specific effect in each individual situation. However, the overall similarity of the results also supports the conclusion that the results from the simulator study are valid and future studies can also be conducted and interpreted in the simulator.

### Limitations

The results could show that it is possible to help passengers in uncomfortable situations. However, the transfer of the results to the effect in daily life is only possible to a limited extent. It must be taken into account when considering the assistance system evaluations by the participants in the post-inquiry that the density of uncomfortable situations per trip was probably higher compared to regular everyday driving and the selection of possible situations was limited by safety considerations. Therefore, these results show more a positive attitude toward these systems rather than a prediction of the ratings for a system on the market in general.

The effect of these assistance systems and its modulation by the relationship between driver and passenger could not be investigated in this study as driver and passenger were not familiar. The responses of the passengers in the post-survey regarding positive and negative aspects of the assistance systems and the fact, that many uncomfortable rides seem to take place with a more familiar driver indicate that this should be looked at in more detail in future studies.

The study was conducted on real roads to increase the external validity of the results. However, this implies that several factors like weather conditions or surrounding traffic could not be controlled. It is possible that these external factors influence the participants’ perception of the test situations and therefore the acceptance ratings.

### Conclusion and Future Research

The user study shows that it is possible to take passenger needs such as comfort into account when designing assistance systems for vehicles by using existing information of other ADAS. It also shows that it is possible to significantly reduce passenger discomfort with systems that display information about the driver’s cognitive state or allow the passenger to have more influence on driving situations. For example, information that is normally only available to the driver can also be made available to the passenger. It is also possible to provide other information for passengers than the information tested in this work. The cognitive co-driver discomfort model can provide a basis for other cognitive states of the driver relevant to the discomfort of the passenger.

Since the driver will slowly transform into a passenger with increasing automation levels in the future, these results can also be relevant to this area. The results could mean that drivers should be able to request information from assistance systems even in automation level 4/5 (SAE International, 2014). They also suggest that for certain drivers it can have a negative impact on their driving experience not being able to intervene in the driving task, as in level 5 (SAE International, 2014).

In general, passengers and their needs should be included in the development of assistance systems. Although, the passenger has been neglected in previous research, the responses of participants of the presented study showed a large interest in these kinds of systems. Further research in this direction might therefore be relevant both for the scientific community as well as for future vehicle applications.

## Data Availability Statement

The raw data supporting the conclusions of this article will be made available by the authors, without undue reservation.

## Ethics Statement

The studies involving human participants were reviewed and approved by the institutional ethics committee at the WIVW GmbH. The patients/participants provided their written informed consent to participate in this study.

## Author Contributions

SI, DM, and TW: conceptualization, methodology, and validation. SI: software, formal analysis, investigation, data curation, writing–original draft preparation, and visualization. SI, TW, and AN: resources and project administration. DM and TW: writing–review and editing. All authors have read and agreed to the published version of the manuscript.

## Funding

This project is part of a research program funded and supported by the Honda Research Institute Europe GmbH.

## Conflict of Interest

This study was conducted as part of a research program of the Honda Research Institute Europe GmbH. TW is employee of this company. He contributed to the design of the study, review and editing of the manuscript, and the decision to publish the results. SI and DM are employed by Wuerzburg Institute for Traffic Sciences GmbH. AN is the managing director of it.

## Publisher’s Note

All claims expressed in this article are solely those of the authors and do not necessarily represent those of their affiliated organizations, or those of the publisher, the editors and the reviewers. Any product that may be evaluated in this article, or claim that may be made by its manufacturer, is not guaranteed or endorsed by the publisher.
