# Supplementary material for: User Evaluation of Passenger Assistance System Concepts on Public Highways
Source: Front Psychol. 2021 Dec 9;12:725808. doi: 10.3389/fpsyg.2021.725808 (PMC8696277; doi:10.3389/fpsyg.2021.725808)
Supplement: Supplementary file 1 [file Table_1.docx]

**Table A. 1**

Frequency of positive and negative statements about the SDAT system made in the post inquiry

| **Positive Statements** | **43** | **Negative Statements** | **22** |
| --- | --- | --- | --- |
| **Driver's attention visible** | **13** | **Not helpful or not useful** | **7** |
| "It's good that you could see that the driver was paying attention" | 13 | "Not necessary for everyday life"  "Distracting"  "Can lead to dispute" | 4  2  1 |
| **Helpful (influence on comfort)** | **10** | **Reliability questionable** | **6** |
| "Calming feeling"  "Feeling in control of the situation"  "Avoids discussions" | 8  1  1 | "Looks not an indicator of attention"  "Maybe too insensitive"  "Varies depending on driver's driving style" | 3  2  1 |
| **Display type (position, visibility, colors)** | **8** | **Display type (position, visibility, colors)** | **6** |
| "Color system easy to understand"  "Position good"  "Good addition" | 3  4  1 | "Not noticeable enough"  "Always looking, you become a control freak"  "System is too fine-tuned" | 4  1  1 |
| **Neutral or objective** | **4** | **Unrealistic or not feasible** | **1** |
| "Objective measure of driver's fitness to drive"  "Helpful with strangers" | 3  1 | "Unrealistic such a system in a car" | 1 |
|  |  |  |  |
| **Useful in a commercial context** | **3** | **Nothing positive** | **2** |
| "Maybe more in a commercial context"  "Good for trucks or frequent drivers" | 2  1 |  |  |
| **Nothing negative** | **5** |  |  |

**Table A. 2**

Frequency of positive and negative statements about the SSD system made in the post inquiry

| **Positive Statements** | **42** | **Negative Statements** | **19** |
| --- | --- | --- | --- |
| **Helpful or provides safety** | **11** | **Not helpful or not necessary** | **6** |
| "You feel safer"  "More attentive in the situation" | 7  4 | "Too insensitive to help passenger"  "Better without it. In what way is it a supplement? (ACC)"  "Vehicle detection unnecessary (white on green change)" | 3  2  1 |
| **Comparison to subjective sensation (objective)** | **11** | **Distance too close to other vehicles** | **6** |
| "Objective measure is good"  "You can compare your own subjective view" | 7  4 | "Too little distance before orange state comes" | 6 |
|  |  |  |  |
| **Distance visible** | **11** | **Display type (position, visibility, colors)** | **3** |
| "Passenger also gets distance" | 11 | "Difficult to see in brightness"  "Distracting"  "Too far away" | 1  1  1 |
|  |  |  |  |
| **Display type (position, visibility, colors)** | **7** | **Familiarization effect** | **2** |
| "Good perceptibility and visibility"  "Simple to understand due to color coding" | 6  1 | "You get used to the system" | 2 |
|  |  |  |  |
| **Nothing negative** | **2** | **Other** | **2** |
|  |  | "Can't see any road users shearing across"  "System confusing" | 1  1 |

**Table A. 3**

Frequency of positive and negative statements about the ASSD system made in the post inquiry

| **Positive Statements** | **36** | **Negative Statements** | **15** |
| --- | --- | --- | --- |
| **Distance displayed (objective)** | **12** | **Needs overcoming to press button (inhibition threshold)** | **5** |
| "Distance is displayed and can be better estimated"  "Visual communication with driver and passenger" | 9  3 | "Overcoming button to press"  "Conflict potential" | 3  2 |
| **Helpful or provides safety** | **10** | **Distance too small** | **4** |
| "Feels more comfortable with it"  "Button gives even more control"  "Both more attentive" | 6  3  1 | "System makes more green – I am ambivalent" | 4 |
|  |  |  |  |
| **Feedback to drivers** | **7** | **Unnecessary or rather verbal feedback** | **3** |
| "Giving the driver feedback without distracting him" | 7 | "Would rather do it orally"  "Doesn't matter, SD is enough" | 2  1 |
|  |  |  |  |
| **Other** | **2** | **Display type (position, visibility, colors)** | **3** |
| "Passengers are different"  "More likely to use in cab" | 1  1 | "Cluttered, distracting"  "Orange not clearly enough visible" | 2  1 |
| **Nothing negative** | **5** |  |  |
